# Supplementary material for: The lived and living experiences of having chronic pain and mental illness among Canadian veterans: A qualitative descriptive study
Source: PLOS Ment Health. 2026 Apr 2;3(4):e0000550. doi: 10.1371/journal.pmen.0000550 (PMC13046254; doi:10.1371/journal.pmen.0000550)
Supplement: S1 Data — (DOCX) [file pmen.0000550.s001.docx]

**Chronic Pain and Mental Health**

How many said there is a real pain relationship? 19

How many said there is no relationship? One only – 8173

**There is no relationship between chronic pain and mental health**

- However, the participant does not think there was a relationship. He believes that the chronic pain developed mostly from injuries and repetitive strains. (8173-wear and tear)

**Mental health affects chronic pain**

- Mental health (PTSD, anxiety disorder, Tourette’s) came before chronic pain diagnosis. (8173-wear and tear)
- Someone from the VA suggested asking for mental health diagnoses in the appeal in addition to TMJ because she was told that physical injuries are often caused by mental health issues. (6866-wear and tear)
- Participant thinks that physical pain is manifestations of mental health challenges. Physical pain should be treated and not ignored. (6866-wear and tear)
- PTSD and chronic pain increased together when she stopped in life to realize and address the issues. (7864-wear and tear)
- The most effective was ketamine treatment for PTSD: “and my brain started to function a lot better. I mean, I’m still not, ‘normal,’ but it’s way better than where I was, and the pain came down with it…So to me, they’re definitely connected. They started at the same time, they reduced at the same time.” (7864-wear and tear)
- She was told that stress from PTSD releases cortisol, which gets trapped in muscles and joints that leads to chronic pain. She does not know why they haven’t been able to figure this out yet and how to manage or treat it. (7864-wear and tear)
- She also was told about the muscle memory theory that when there is pain, the body remembers that pain and that is the future cause of pain. She doesn’t believe in this as much for her pain, while she agrees that some people have this kind of pain. Her concern is that this theory is pushed to all kinds of pain, and this leads to less effective and compassionate treatment. She believes her pain is there and ignoring or distracting from that does not help because it is always constant pain: “because this theory is being pushed more, we’re being ignored, again, marginalized, because it’s just in my head.” (7864-wear and tear)
- “And I think the easy answer sometimes is going to that part of it’s just in your head…and if they don’t understand what it feels like, then it makes it hader for them to be compassionate to that…because there’s more and more studies coming out that it is just mental, you know, it’s motivating for them to use it, to use that line.” (7864-wear and tear)
- PTSD impact on chronic pain: “I would say that it went up because I like when I get anxious, or any of that, I like, I hold that energy in and it manifests itself in me not looking after myself.” (1652-injury and no release)
- “but what I thought would be normal of just missing my family because, you know, they're in [city], and I'm in [city], that I was more depressed. I was not energetic. I was very lethargic. I, you know, all of that kind of stuff. So when you're not moving, you know, it you're all of your injuries just become accentuated.” (1652-injury and no release)
- It is easier to deal with the physical pain but much harder to deal with mental pain and health: “it makes it harder to work through things mentally. It's working through the physical is easy. You know, I got a cane sitting over here in the corner now, I mean that I use regularly, and coming to grips with that, it certainly at only 64 I don't appreciate it, I mean, because I know guys still running at my age, but it's easier than coming to grips with how it influences my my mental health.” (4993-injury and no release)
- At a vacation, he could not go with his wife walking outside to explore the city and told wife to go alone but that took a toll on him and his identity as a veteran and older person: “It was mentally hard sitting back there knowing that I wanted to be out there, just would have just held her back. That's the hardest part of it is, you know, like, like not feeling like and go for a run, for one I mean, I used to run, even in the later days.” (4993-injury and no release)
- Going to the store can cause back pain due to the stress from the crowd (9443-injury without release)
- Stress from work caused more back pain: “I still had that stress, like, I have to be there, I can’t be late, I have to do this, you know, all that military mindset, the “I need to perform” mentality. It has to show results. All of that just increased my anxiety. I had more back pain, more ankle pain, headaches. The days I went there, I’d get headaches. The days I had to go there, I’d just think, "Wow, this is crazy." It’s crazy how connected everything is” (9443-injury without release)
- He held a lot of resentment for the bus driver that cut him off and caused his accident, which made it hard to mentally move on. He now has come to peace with it and doesn’t “dwell on the bus driver. My focus is on the kids we saved, That’s what matters now” (referring to the kids in the school bus) (8262-injury without release)
- He explained how his pain fluctuates with his mental state. “When I feel relaxed and things are going well, I don’t feel pain. But as soon as stress creeps in, I feel it—especially when I struggle to do something as simple as walking around the block with my dog.” He finds this particularly frustrating and compounds the mental toll of PTSD. (8262-injury without release)
- Chronic pain also creates stress for him when he cannot complete tasks that others take for granted. He described this as a recurring challenge in his daily life. Tasks include walking his dog, cooking, cleaning, doing laundry, etc. (8262-injury without release)
- Has been through mental health programs which he feels helps him put less pressure on himself and better understand himself. This also helped him learn to put less pressure on his performance and physical abilities. (8262-injury without release)

**Chronic pain affects mental health**

- “My mental health never stopped me from fixing my physical health, but my physical health was a huge factor in how much success I could have in dealing with my mental health.” (3528-wear and tear)
- “The physical pain affected my mental health. Like affected how confident you feel, how fearful you are. Like, there was a long time I never felt safe. I didn’t feel safe about how I was perceived. I didn’t feel safe about if something bad were to happen, would I have the strength and physical capability to deal with that situation?” (3528-wear and tear)
- Gabapentin treatment for his chronic pain caused him to have suicidal thoughts that led to him getting therapy and getting a PTSD diagnosis. The PTSD diagnosis was really important for him: “So that disjointedness, that lack follow up, had been hiding mental problems, and once the doctor, my psychologist, explained to me that I had PTSD, all of the things, all of the pennies dropped into place, the lights went on, I said, Now I know, and now I understand. And my life became clear.” (2791-injury and no release)
- He describes how the chronic pain makes his mental health worse. Whenever he is doing work around the house, his pain gets worse, which makes his mental health worse. (3735-injury and release)
- The chronic pain made him wake up in the middle of the night or not sleep at all. Because of its severity, it took pills but when he did, a whole host of emotions, feelings, and mental pain came related to his PTSD: “it just seemed like the chronic pain compounded my mental health. My mental health didn’t help my chronic pain, and it was just a big circle.” (7139-injury and no release)
- “you have all these experiences, and I put them in a pack sock and carry on. I go to so much, both from the military and Red Cross, but from the military. You put them away and you carry them. You let them out Remembrance Day. I let them out at different times. You think about them. You pack them up, put them back in your pack and zip it up and carry on. I just found as the chronic pain got worse, it's almost like the zipper broke and things would fall out when I didn't want it to fog, and then suddenly I'd be in traffic, or suddenly I'd be with my partner, and the sights and sounds or the smells would just trigger me.” (7139-injury and no release)
- “You get fed up with dealing with that pain all the time, like, it just wears you down so much. And there’s a lot of things in your life that you know you just cant do anymore, or you’re markedly restricted from being able to do, like grocery and standing in line for just to pay your groceries…” (4119-injury and release)
- Chronic pain makes life more difficult including mental health, especially the loss of identity from having to leave military service and go into a different world, the civilian life. (4119-wear and tear)
- Chronic pain drains ability to enjoy things and it makes mental health a lot worse. (4119-wear and tear)
- “And I think dealing with the chronic pain and that the chronic pain constantly wears on you, and I think that's another thing that really people don't understand, is how badly it just saps your energy because you're fighting every day. It's constant. So that takes its toll emotionally on you, because it not only impacts your physical well being, but your emotional well being, of things that you used to be able to do you can't do now, and trying to get into the old things that you used to be able to do that you used to enjoy?” (4119-wear and tear)
- “You can barely go for a walk. We go for a walk, and we're about five minutes into the walk, and he's in pain, and we have to turn around.” (4119-wear and tear)
- “And it's just, it's you find it draining, and it sort of, it grates on your ability to be able to enjoy things. It makes your depression even worse, because now you're also dealing with the pain you're dealing with your depression. It's also tied in with your loss of self and everything after being taken, you know, being moved out of the military. You know, I, I joined when I was 18. So, I mean, that's the only thing I'd I'd known for majority of my life. I spent more time in the military than I did as a civilian. So, you know, you get ingrained into that mindset, that mentality of how to conduct yourself, on how things are done.” (4119-wear and tear)
- The spiral downward of chronic pain: “because that chronic pain drains your energy, it drains your ability to do things, and then that just feeds on top of the depression, the PTSD relationships start to suffer. And then you turn into that, that inside your head spiral, and that's that gets hard to get out of. And then you start isolating yourself more, because, well, every time I go out, people bitch I'm too loud. People bitch on this and that fine, I just won't freaking go. And then pretty soon that whole social circle, or your social environment, gets smaller, smaller, smaller, and pretty soon it's just you and your house. And that's where I was getting to.” (4119-wear and tear)
- She has regular tension headaches that are caused by chronic pain state, but these headaches started during military service: “It just depends on when my chronic pain is up, then my tension seems to be up, and then my headache seemed to be up. So I think it's all interconnected there, but it just depends. Like, some days it's debilitating to the point where I just have, I can't look at screens. I'm just like, Okay, enough of this. I'm just going to go up do something else, you know, kind of shake it off so that you can, you know, get away from whatever you're doing, and then kind of hit the reset button, kind of thing to come back at something later.” (1652-injury and no release)
- He has to deal with his pain first, then PTSD, everday: “As you can see from the two examples, the two injuries (chronic pain and PTSD) work together and feed off of each other and are deeply connected. For me, I have found that in order to be able to effectively address my PTSD issues, I must first deal with my chronic pain issues each day. The minute that my feet hit the ground in the morning I'm thinking about my pain levels and how to maintain or improve them so that I have a good day from a chronic pain standpoint. Once I've started the work in dealing with my chronic pain then I can get busy with the work of dealing with my PTSD by using all of the tools in my toolbox. In order to deal with chronic pain and PTSD you have to put in the time and do the work each day.” (6150-injury and release)
- Did a session at St. Anne Hospital in 2024 to explore the link between chronic pain and mental health: “They explained to us in this course that you absolutely have to keep moving. Because, either way, whether you move or not, it’s going to hurt, OK? But the thing is, when you move at that moment… It actually helps your body more on the mental health side. It’s going to make you feel better, for sure, it’s going to help, and little by little, while you’re doing your exercises, you’ll think less about your pain.” (9443-injury without release)

**Both chronic pain and mental health affect each other**

- PTSD affects chronic pain. One can exacerbate the other. Because he is in so much pain, there is limited mental energy to act normal. (4050-injury without release)
- He believes his chronic pain and depression go hand in hand “if you didn’t have this pain, you would not have the depression…living with the pain every day, it just became overwhelming.” (6009-injury and release)
- Doing the treatments to reduce his chronic pain allowed him to get out of depression: “But her treatments have brought me out of that depressive stage, you know, whereas, right now, I feel like I can get on with my life and I can do things.” (6009-injury and release)
- “You know, the mind says, yeah, you can do this, do this, do this. But the body is going, well, hang on a second here.” (8775-injury and no release)
- He believed that they are intertwined but he is still exploring to the extent: “it’s kind of a catch 22. You’re in pain. You can’t sleep. You can’t sleep. They can’t treat the PTSD. They can’t treat the PTSD. You get frustrated or depressed and you feel the pain more.” (8775-injury and no release)
- “When one is good, the other one is good too. When one is bad, it can bring the other one down as well.” (3735-injury and release)
- He compartmentalizes it but the connection between mental and physical health helps him: “I think the physical injuries are what they are, and I try and compartmentalize it that way. It's just when things happen like what happened in Montenegro, it spills over a little bit, and I just have to take a breath and find find a place. Oh, it's certainly I don't think. I think if I didn't have the foundational knowledge that I do, it would be worse. I think if I didn't understand why I feel so alone and distanced and ostracized, if I didn't understand that and the connection to my physical limitations, you know, scientifically, I think would make it a lot harder, but no, I mean I understanding besides where it comes from and how it evolves and what I'm dealing with, I think helps.” (4993-injury and no release)
- Both are connected but he has days where PTSD is good but if pain is severe, then the PTSD gets triggered. Furthermore, the opposite is also true when chronic pain is good but PTSD is not and the pain is then triggered. He has recorded over 18 different types of triggers for PTSD. (6150-injury and release)

**Pain limits energy levels, which limits activities, which exacerbates mental health**

- PTSD affects chronic pain. One can exacerbate the other. Because he is in so much pain, there is limited mental energy to act normal. (4050-injury without release)
- “The physical pain affected my mental health. Like affected how confident you feel, how fearful you are. Like, there was a long time I never felt safe. I didn’t feel safe about how I was perceived. I didn’t feel safe about if something bad were to happen, would I have the strength and physical capability to deal with that situation?” (3528-wear and tear)
- “You get fed up with dealing with that pain all the time, like, it just wears you down so much. And there’s a lot of things in your life that you know you just cant do anymore, or you’re markedly restricted from being able to do, like grocery and standing in line for just to pay your groceries…” (4119-injury and release)
- Chronic pain drains ability to enjoy things and it makes mental health a lot worse. (4119-wear and tear)
- “And I think dealing with the chronic pain and that the chronic pain constantly wears on you, and I think that's another thing that really people don't understand, is how badly it just saps your energy because you're fighting every day. It's constant. So that takes its toll emotionally on you, because it not only impacts your physical well being, but your emotional well being, of things that you used to be able to do you can't do now, and trying to get into the old things that you used to be able to do that you used to enjoy?” (4119-wear and tear)
- “You can barely go for a walk. We go for a walk, and we're about five minutes into the walk, and he's in pain, and we have to turn around.” (4119-wear and tear)
- “And it's just, it's you find it draining, and it sort of, it grates on your ability to be able to enjoy things. It makes your depression even worse, because now you're also dealing with the pain you're dealing with your depression. It's also tied in with your loss of self and everything after being taken, you know, being moved out of the military. You know, I, I joined when I was 18. So, I mean, that's the only thing I'd I'd known for majority of my life. I spent more time in the military than I did as a civilian. So, you know, you get ingrained into that mindset, that mentality of how to conduct yourself, on how things are done.” (4119-wear and tear)
- The spiral downward of chronic pain: “because that chronic pain drains your energy, it drains your ability to do things, and then that just feeds on top of the depression, the PTSD relationships start to suffer. And then you turn into that, that inside your head spiral, and that's that gets hard to get out of. And then you start isolating yourself more, because, well, every time I go out, people bitch I'm too loud. People bitch on this and that fine, I just won't freaking go. And then pretty soon that whole social circle, or your social environment, gets smaller, smaller, smaller, and pretty soon it's just you and your house. And that's where I was getting to.” (4119-wear and tear)

**Physical health prevented getting help because of pain**

- “My mental health never stopped me from fixing my physical health, but my physical health was a huge factor in how much success I could have in dealing with my mental health.” (3528-wear and tear)
- He has to deal with his pain first, then PTSD, everday: “As you can see from the two examples, the two injuries (chronic pain and PTSD) work together and feed off of each other and are deeply connected. For me, I have found that in order to be able to effectively address my PTSD issues, I must first deal with my chronic pain issues each day. The minute that my feet hit the ground in the morning I'm thinking about my pain levels and how to maintain or improve them so that I have a good day from a chronic pain standpoint. Once I've started the work in dealing with my chronic pain then I can get busy with the work of dealing with my PTSD by using all of the tools in my toolbox. In order to deal with chronic pain and PTSD you have to put in the time and do the work each day.” (6150-injury and release)
- Did a session at St. Anne Hospital in 2024 to explore the link between chronic pain and mental health: “They explained to us in this course that you absolutely have to keep moving. Because, either way, whether you move or not, it’s going to hurt, OK? But the thing is, when you move at that moment… It actually helps your body more on the mental health side. It’s going to make you feel better, for sure, it’s going to help, and little by little, while you’re doing your exercises, you’ll think less about your pain.” (9443-injury without release)

**Pain treatment exacerbated underlying mental health issues**

- Gabapentin treatment for his chronic pain caused him to have suicidal thoughts that led to him getting therapy and getting a PTSD diagnosis. The PTSD diagnosis was really important for him: “So that disjointedness, that lack follow up, had been hiding mental problems, and once the doctor, my psychologist, explained to me that I had PTSD, all of the things, all of the pennies dropped into place, the lights went on, I said, Now I know, and now I understand. And my life became clear.” (2791-injury and no release)
- The chronic pain made him wake up in the middle of the night or not sleep at all. Because of its severity, it took pills but when he did, a whole host of emotions, feelings, and mental pain came related to his PTSD: “it just seemed like the chronic pain compounded my mental health. My mental health didn’t help my chronic pain, and it was just a big circle.” (7139-injury and no release)

**Stressful situations increases pain**

- Going to the store can cause back pain due to the stress from the crowd (9443-injury without release)
- Stress from work caused more back pain: “I still had that stress, like, I have to be there, I can’t be late, I have to do this, you know, all that military mindset, the “I need to perform” mentality. It has to show results. All of that just increased my anxiety. I had more back pain, more ankle pain, headaches. The days I went there, I’d get headaches. The days I had to go there, I’d just think, "Wow, this is crazy." It’s crazy how connected everything is” (9443-injury without release)
- He held a lot of resentment for the bus driver that cut him off and caused his accident, which made it hard to mentally move on. He now has come to peace with it and doesn’t “dwell on the bus driver. My focus is on the kids we saved, That’s what matters now” (referring to the kids in the school bus) (8262-injury without release)
- He explained how his pain fluctuates with his mental state. “When I feel relaxed and things are going well, I don’t feel pain. But as soon as stress creeps in, I feel it—especially when I struggle to do something as simple as walking around the block with my dog.” He finds this particularly frustrating and compounds the mental toll of PTSD. (8262-injury without release)
- Chronic pain also creates stress for him when he cannot complete tasks that others take for granted. He described this as a recurring challenge in his daily life. Tasks include walking his dog, cooking, cleaning, doing laundry, etc. (8262-injury without release)
- Has been through mental health programs which he feels helps him put less pressure on himself and better understand himself. This also helped him learn to put less pressure on his performance and physical abilities. (8262-injury without release)

**Mental health reduced confidence to seek help, get treatment or participate in activities**

- PTSD impact on chronic pain: “I would say that it went up because I like when I get anxious, or any of that, I like, I hold that energy in and it manifests itself in me not looking after myself.” (1652-injury and no release)
- “but what I thought would be normal of just missing my family because, you know, they're in [city], and I'm in [city], that I was more depressed. I was not energetic. I was very lethargic. I, you know, all of that kind of stuff. So when you're not moving, you know, it you're all of your injuries just become accentuated.” (1652-injury and no release)
- It is easier to deal with the physical pain but much harder to deal with mental pain and health: “it makes it harder to work through things mentally. It's working through the physical is easy. You know, I got a cane sitting over here in the corner now, I mean that I use regularly, and coming to grips with that, it certainly at only 64 I don't appreciate it, I mean, because I know guys still running at my age, but it's easier than coming to grips with how it influences my my mental health.” (4993-injury and no release)
- At a vacation, he could not go with his wife walking outside to explore the city and told wife to go alone but that took a toll on him and his identity as a veteran and older person: “It was mentally hard sitting back there knowing that I wanted to be out there, just would have just held her back. That's the hardest part of it is, you know, like, like not feeling like and go for a run, for one I mean, I used to run, even in the later days.” (4993-injury and no release)

**Mental health diagnosis serves as clarity and explanation**

- Mental health diagnoses has not impacted pain level but explained why he thinks the way he thinks. Mental health diagnoses has given him confidence that he was receiving help and that the VA will finally look at him properly. He can’t explain this much. (7149-injury and release)
- Someone from the VA suggested asking for mental health diagnoses in the appeal in addition to TMJ because she was told that physical injuries are often caused by mental health issues. (6866-wear and tear)
- PTSD and chronic pain increased together when she stopped in life to realize and address the issues. (7864-wear and tear)
- The most effective was ketamine treatment for PTSD: “and my brain started to function a lot better. I mean, I’m still not, ‘normal,’ but it’s way better than where I was, and the pain came down with it…So to me, they’re definitely connected. They started at the same time, they reduced at the same time.” (7864-wear and tear)
- She was told that stress from PTSD releases cortisol, which gets trapped in muscles and joints that leads to chronic pain. She does not know why they haven’t been able to figure this out yet and how to manage or treat it. (7864-wear and tear)
- She also was told about the muscle memory theory that when there is pain, the body remembers that pain and that is the future cause of pain. She doesn’t believe in this as much for her pain, while she agrees that some people have this kind of pain. Her concern is that this theory is pushed to all kinds of pain, and this leads to less effective and compassionate treatment. She believes her pain is there and ignoring or distracting from that does not help because it is always constant pain: “because this theory is being pushed more, we’re being ignored, again, marginalized, because it’s just in my head.” (7864-wear and tear)
- “And I think the easy answer sometimes is going to that part of it’s just in your head…and if they don’t understand what it feels like, then it makes it hader for them to be compassionate to that…because there’s more and more studies coming out that it is just mental, you know, it’s motivating for them to use it, to use that line.” (7864-wear and tear)
- He compartmentalizes it but the connection between mental and physical health helps him: “I think the physical injuries are what they are, and I try and compartmentalize it that way. It's just when things happen like what happened in Montenegro, it spills over a little bit, and I just have to take a breath and find find a place. Oh, it's certainly I don't think. I think if I didn't have the foundational knowledge that I do, it would be worse. I think if I didn't understand why I feel so alone and distanced and ostracized, if I didn't understand that and the connection to my physical limitations, you know, scientifically, I think would make it a lot harder, but no, I mean I understanding besides where it comes from and how it evolves and what I'm dealing with, I think helps.” (4993-injury and no release)

**Mental health diagnosis gave confidence to receive help**

- Mental health diagnoses has not impacted pain level but explained why he thinks the way he thinks. Mental health diagnoses has given him confidence that he was receiving help and that the VA will finally look at him properly. He can’t explain this much. (7149-injury and release)
- Someone from the VA suggested asking for mental health diagnoses in the appeal in addition to TMJ because she was told that mental health issues often cause physical injuries. (6866-wear and tear)
