# Supplementary material for: The lived and living experiences of having chronic pain and mental illness among Canadian veterans: A qualitative descriptive study
Source: PLOS Ment Health. 2026 Apr 2;3(4):e0000550. doi: 10.1371/journal.pmen.0000550 (PMC13046254; doi:10.1371/journal.pmen.0000550)
Supplement: Attachment — Submitted filename: Response to Peer Reviewers.docx [file pmen.0000550.s003.docx]

**Response to Peer-Reviewers**

December 30, 2025

| **Comment** | **Response** | **Page #** |
| --- | --- | --- |
| Journal Requirements | | |
| In the ethics statement in the Methods, you have specified that verbal consent was obtained. Please provide additional details regarding how this consent was documented and witnessed, and state whether this was approved by the IRB. | We have added additional clarity to the consent process.  “The University of Toronto Research Ethics Board approved this study. All participants provided written consent via email or verbal consent on recording before the interview started and this procedure was approved by the University of Toronto Research Ethics Board.” | 10 |
| Reviewer 1 | | |
| This is an engaging and methodologically sound qualitative manuscript that provides an important contribution to understanding the intersection of chronic pain and mental illness among Canadian Veterans. The integration of participants’ narratives with theoretical models adds considerable depth and clinical relevance. The following suggestions are intended to strengthen an already strong manuscript. | Thank you for these positive comments, and for taking the time to read and provide additional feedback. | NA |
| Major Strengths - The study captures rich, authentic narratives that humanize Veterans’ lived and living experiences. - The theoretical framing is thoughtful and multidimensional, bridging biomedical, psychological, and sociocultural perspectives. - The methodological rigor is evident, and the inclusion of a Veteran partner in the analysis process is a notable strength. - The findings are highly relevant to integrated and trauma-informed care approaches for Veterans. | Thank you for highlighting the strengths of our paper. We appreciate the time you’ve taken to review our paper and provide feedback to further strengthen the paper. | NA |
| - Clarify Theoretical Integration: Explicitly differentiate how the Shared Vulnerability and Mutual Maintenance models each uniquely apply to Veterans’ experiences. | Thank you for this suggestion. We have further clarified the two models and how they uniquely apply to Veterans.  “**While the Shared Vulnerability model emphasizes** pre-existing, shared etiological mechanisms **that increase Veterans’ risk for developing both chronic pain and mental illness, the Mutual Maintenance model highlights the** ongoing, reciprocal processes **through which these conditions might interact and reinforce one another once established. This distinction is particularly salient for Veterans whose trauma exposure and injury may both initiate comorbidity and perpetuate symptoms through daily lived and living experiences.”** | 5 |
| - Expand Intersectional Discussion: Consider acknowledging gendered and racialized aspects of the sample (85% White, 75% male) to situate findings within broader intersectional contexts. | Thank you for this comment. We have added intersectional discussion in the discussion section and throughout the paper. We have also added this as a limitation in the limitations section.  “Second, the sample was predominantly composed of White male Veterans. Experiences of comorbidity are likely shaped by intersecting forms of marginalization, including gender, race, and access to social and health resources, all of which were underrepresented in this study. The findings may not fully capture how the intersection of chronic pain and mental illness is experienced by Veterans across diverse gendered, racialized, and social identities. Future research should prioritize more diverse and purposive sampling strategies to examine how chronic pain and mental illness are experienced within broader social and structural contexts among underrepresented Veteran populations.” | 25 |
| - Reflexivity/positionality: Briefly elaborate on how the Veteran co-researcher’s perspective shaped data interpretation | Thank you for this comment. We have added a new subsection after data analysis discussing the reflexivity of our Veteran partner and how they were engaged in this work.  “**Veteran Partner Reflexivity**  The inclusion of a Veteran partner on the research team brought experiential knowledge of military service, chronic pain, and mental health that informed the interpretation of participants’ accounts. This perspective supported analytic sensitivity to the nuances of Veterans’ language, contextual meanings, and everyday realities that may not be readily apparent to non-Veteran researchers. At the same time, the Veteran partner engaged in ongoing reflexive dialogue with the broader research team to critically examine how shared experiences could shape interpretations.” | 10 |
| - Findings Section: Condense slightly and consider using subhead summaries or a visual model to illustrate the cyclical pain–mental illness feedback loop. | Thank you for this comment. We have added a table to summarize the themes and subthemes of the findings section (two themes and two subthemes each). We believe that this is a relatively simple structure to follow. However, we agree that the content can appear drawn out so we have taken this opportunity to further clarify and condense the material without losing meaning. However, we don’t believe we have sufficient information to create a visual model. This remains an area for future research. | 12 |
| - Discussion: Add a short paragraph on practical implications for Veteran health systems (e.g., integrated care, biopsychosocial rehabilitation, or interdisciplinary treatment). | Thank you for this comment. We have added an implications section to the paper.  “**Implications**  The findings of this study have important implications for Veteran health systems, particularly the need to move beyond siloed, diagnosis-driven models of care toward approaches that recognize chronic pain and mental illness as interdependent and mutually shaping conditions. Veterans’ accounts show that treating pain and mental health in isolation risks overlooking the cyclical, embodied ways these conditions interact to erode identity, functioning, and social connection. Health systems serving Veterans must therefore adopt integrated, trauma-informed models of care that explicitly acknowledge how military service, identity, and transition to civilian life shape both pain and psychological distress. This includes care pathways that enable mental health and pain services to work collaboratively and support the restoration of meaning, agency, and belonging. Veterans’ narratives further suggest that clinical encounters should create space for Veterans to articulate how they understand the origins and interconnections of their pain and mental illness, rather than imposing narrow biomedical explanations that may feel incomplete or invalidating. Incorporating these lived and living perspectives into assessment, care planning, and rehabilitation may help reduce disengagement from services and improve continuity of care. Ultimately, Veteran health systems that foreground the relational, contextual, and identity-based dimensions of comorbidity may be better positioned to support healing that extends beyond symptom reduction to encompass social reintegration and long-term well-being.” | 24-25 |
| - Editorial Polishing: Minor proofreading and clarification (e.g., consistency in tense and citation style) will further strengthen clarity. | Thank you for this comment. We have reviewed the manuscript to proofread and further clarify different parts of the manuscript. We hope the revised version reads better. | NA |
| Reviewer 2 | | |
| The rationale for examining the lived and living experiences of military veterans requires further strengthening. While the topic is relevant, the arguments that build toward this focus remain underdeveloped. It is recommended that the authors elaborate on these points by clearly articulating why such experiences warrant focused scholarly attention and how they contribute to existing knowledge. | Thank you for this suggestion. We have strengthened our rationale for focusing on this topic in the introduction section.  “Much of the existing literature has relied on quantitative or symptom-focused approaches, which, while informative, provide limited insight into how Veterans interpret, manage, and live with this comorbidity on a day-to-day basis [Pratt 2025, Gros 2015, Plagge 2013]. Relatively little qualitative research has explored how Canadian Veterans understand the interconnected nature of chronic pain and mental illness or how these conditions shape their post-service lives within civilian life. Articulating Veterans’ own perspectives addresses a critical gap in the literature by extending theoretical models of comorbidity into lived/living experience and informing more responsive, Veteran-centred care.” | 5-6 |
| Relatedly, the research gap is not sufficiently articulated. The manuscript would benefit from a clearer and more explicit statement of the gap that this study seeks to address, particularly in relation to prior empirical or theoretical work on military veterans’ experiences. | Thank you for this suggestion. We have added additional detail and references to strengthen how we explain the research gap.  “Much of the existing literature has relied on quantitative or symptom-focused approaches, which, while informative, provide limited insight into how Veterans interpret, manage, and live with this comorbidity on a day-to-day basis [Pratt 2025, Gros 2015, Plagge 2013]. Relatively little qualitative research has explored how Canadian Veterans understand the interconnected nature of chronic pain and mental illness or how these conditions shape their post-service lives within civilian life. Articulating Veterans’ own perspectives addresses a critical gap in the literature by extending theoretical models of comorbidity into lived/living experience and informing more responsive, Veteran-centred care.” | 5-6 |
| The central research question is also not clearly evident. To improve coherence and direction, the authors are encouraged to explicitly state the main research question (and/or sub-questions) guiding the inquiry. | Thank you for the suggestion to add clear research questions. We have done so in the revised manuscript at the end of the introduction section.  “The central research questions guiding this study were: how do Canadian Veterans perceive and navigate the relationship between their chronic pain and mental illness? In what ways do these interconnected conditions shape their lived and living experiences in civilian life?” | 6 |
| The study indicates the use of a qualitative descriptive approach; however, the emphasis on participants’ lived and living experiences appears to also be closely aligned with a phenomenological orientation. The authors may wish to reconsider their methodological framing and clarify whether a phenomenological approach should be incorporated or explicitly justified as not being used. | We appreciate the reviewer’s observation about the alignment between lived/living experience and phenomenological inquiry. While phenomenology is a well-established and valuable approach for examining lived experience, it is not the only qualitative methodology for meaningfully exploring lived and living experiences. In this study, we intentionally employed a qualitative descriptive approach to address a focused set of research questions nested within a broader narrative inquiry. The qualitative descriptive orientation was selected to remain close to participants’ accounts and to provide a pragmatic, low-inference description of how Veterans understand and navigate the intersection of chronic pain and mental illness in everyday life. Incorporating a phenomenological approach would have required a distinct methodological framing, analytic strategy, and philosophical orientation that were not aligned with the aims or design of the parent narrative inquiry study. As such, embedding phenomenology within this project would not have been methodologically appropriate and would have necessitated a separate, standalone research endeavor. | NA |
| Further clarification is needed regarding participant selection. The inclusion and exclusion criteria should be clearly defined, and the manuscript should specify how participants were recruited or accessed. Providing this information will enhance the transparency and rigor of the study. | We agree that we were unclear in the first draft about recruitment and sampling. We have revised the section to add additional clarity, and we hope this addresses your comments.  “All Veterans who participated in the broader narrative inquiry study answered questions related to the intersection between chronic pain and mental illness that informed this paper. In the broader narrative inquiry study, eligible participants were Canadian Veterans aged 18 or older with chronic pain who had previously agreed to share their experiences of military and civilian life as part of the project. Veterans currently serving in the military and non-Canadian forces were excluded. Recruitment sought a convenience sample of Veterans facilitated through the Chronic Pain Centre of Excellence for Canadian Veterans listserv. An email was sent out to the listserv and interested Veterans reached out directly to the research team to learn more about the study and how they can participate. **The sample size (n = 20) was selected to capture a range of complex and diverse experiences for the broader narrative inquiry study rather than to achieve thematic saturation. This approach was appropriate for the exploratory nature of this study, which aimed to explore variability in how Veterans understand and live with both chronic pain and mental illness, rather than to produce an exhaustive or homogeneous account of these experiences.”** | 8 |
| In addition, the demographic characteristics of the participants are not adequately presented in the manuscript but was only presented in a table as part of the appendices. A clearer presentation of relevant demographic information, such as age range, length of service, rank, or other pertinent characteristics, as they would help contextualize the findings. | Thank you for this comment. Due to manuscript policies, we attached the table of demographic and social identity characteristics as a separate file, but it is intended to be included within the manuscript upon publication.  We did not integrate additional social identities or military characteristics into the analytic presentation or link them directly to participant quotes for ethical reasons. Given the nature of the stories and experiences shared, the quotes are highly identifiable, and as outlined in our Research Ethics Board guidelines, linking additional social identity or military characteristics to these narratives could increase the risk of participant identification. To balance transparency with participant protection, we therefore opted to provide a high-level summary of participant characteristics in table format rather than attributing detailed identifiers to individual quotations. We hope this approach appropriately addresses both the reviewer’s concern and our ethical obligations. |  |
| It is also important to note that military veterans may be considered a vulnerable population, particularly given the sensitive nature of the topics discussed, such as bodily pain and its connection to mental health concerns. The manuscript would be strengthened by explicitly addressing the potential risks involved and detailing the measures taken to mitigate these risks during data collection, such as ethical safeguards, referral mechanisms, or support provisions. | This is a really important point and we are glad you brought this up. We have added a short paragraph in our data collection section discussing how we safeguarding against moments of distress or emotional discomfort.  “Given that discussions of chronic pain and mental health evoked distressing memories or emotional discomfort, several ethical safeguards were embedded throughout the data collection process. Before participation, Veterans were informed of the sensitive nature of the interview topics and reminded that participation was voluntary, that they could decline to answer any question, and that they could pause or withdraw from the interview at any time without consequence. Interviewers were attentive to verbal and non-verbal cues of distress and were prepared to slow the pace, redirect the conversation, take breaks, or stop the interview if needed. When participants expressed emotional discomfort, interviewers responded with empathy and grounding techniques using a trauma-informed interviewing protocol developed by the research team and Veteran partner, and participants were offered breaks or the option to reschedule. All participants were provided with information about mental health and Veteran-specific support resources at the conclusion of the interview, including instructions on how to access additional support if distress emerged after participation. These measures were implemented to minimize potential risks and to support participants’ well-being throughout the research process.” | 9 |
| Finally, the study currently lacks clearly articulated limitations and recommendations. It is recommended that these sections be added after the Discussion to acknowledge the study’s constraints and to offer directions for future research and practice. | Thank you for this comment. We agree a section on implications and limitations is important. We have included them in the revised version of manuscript. | 24-25 |
| Reviewer 3 | | |
| Abstract: The abstract is well written. | Thank you for the positive comments. | NA |
| Introduction: The introduction is also well elaborated, clearly showing the interplay between chronic pain and mental illness | Thank you for the positive comments. | NA |
| Methods: Elaborate more on how sampling procedure of the study participants (was it purposive or was it convenience sampling) | We agree that we were unclear in the first draft about recruitment and sampling. We have revised the section to add additional clarity, and we hope this addresses your comments.  “All Veterans who participated in the broader narrative inquiry study answered questions related to the intersection between chronic pain and mental illness that informed this paper. In the broader narrative inquiry study, eligible participants were Canadian Veterans aged 18 or older with chronic pain who had previously agreed to share their experiences of military and civilian life as part of the project. Veterans currently serving in the military and non-Canadian forces were excluded. Recruitment sought a convenience sample of Veterans facilitated through the Chronic Pain Centre of Excellence for Canadian Veterans listserv. An email was sent out to the listserv and interested Veterans reached out directly to the research team to learn more about the study and how they can participate. **The sample size (n = 20) was selected to capture a range of complex and diverse experiences for the broader narrative inquiry study rather than to achieve thematic saturation. This approach was appropriate for the exploratory nature of this study, which aimed to explore variability in how Veterans understand and live with both chronic pain and mental illness, rather than to produce an exhaustive or homogeneous account of these experiences.”** | 8 |
| Clarify how the sample size of 20 participants was determined. (What guided you to use 20 study participants and not any other number?) |  |  |
| Sample and Recruitment: First, state the inclusion criteria for the broader narrative inquiry study focusing on identity in Canadian Veterans with chronic pain. Then follow this with what you considered to recruit participants in this current study. |  |  |
| Data collection: Asking veterans about the interplay between their mental illness and chronic pain might evoke painful memories during the interviews. How were such situations handled during your interviews? | This is a really important point and we are glad you brought this up. We have added a short paragraph in our data collection section discussing how we safeguarding against moments of distress or emotional discomfort.  “Given that discussions of chronic pain and mental health evoked distressing memories or emotional discomfort, several ethical safeguards were embedded throughout the data collection process. Before participation, Veterans were informed of the sensitive nature of the interview topics and reminded that participation was voluntary, that they could decline to answer any question, and that they could pause or withdraw from the interview at any time without consequence. Interviewers were attentive to verbal and non-verbal cues of distress and were prepared to slow the pace, redirect the conversation, take breaks, or stop the interview if needed. When participants expressed emotional discomfort, interviewers responded with empathy and grounding techniques using a trauma-informed interviewing protocol developed by the research team and Veteran partner, and participants were offered breaks or the option to reschedule. All participants were provided with information about mental health and Veteran-specific support resources at the conclusion of the interview, including instructions on how to access additional support if distress emerged after participation. These measures were implemented to minimize potential risks and to support participants’ well-being throughout the research process.” | 9 |
| Findings: Write the abbreviation VAC in full (for a reader to understand better) | Thank you for noting this. We have removed VAC and replaced it with a government agency. | 12 |
| In the Demographic table of the participants, Migraines & Conflict morality are listed as examples of Mental Illness Diagnoses. Could you clarify more on these two diagnoses | We have clarified migraines and conflict morality in the demographic table. | Separate table. |
| Discussion: The findings are well discussed, comparing and critiquing the existing literature. | Thank you for the positive comments. |  |
